# Releasing N-glycan from Peptide N-terminus by N-terminal Succinylation Assisted Enzymatic Deglycosylation

**DOI:** 10.1038/srep09770

**Published:** 2015-04-22

**Authors:** Yejing Weng, Zhigang Sui, Hao Jiang, Yichu Shan, Lingfan Chen, Shen Zhang, Lihua Zhang, Yukui Zhang

**Affiliations:** 1 Key Lab of Separation Sciences for Analytical Chemistry, National Chromatographic R. & A. Center, Dalian Institute of Chemical Physics, Chinese Academy of Sciences, Dalian 116023, China; 2 University of Chinese Academy of Sciences, Beijing 100049, China

## Abstract

Due to the important roles of N-glycoproteins in various biological processes, the global N-glycoproteome analysis has been paid much attention. However, by current strategies for N-glycoproteome profiling, peptides with glycosylated Asn at N-terminus (PGANs), generated by protease digestion, could hardly be identified, due to the poor deglycosylation capacity by enzymes. However, theoretically, PGANs occupy 10% of N-glycopeptides in the typical tryptic digests. Therefore, in this study, we developed a novel strategy to identify PGANs by releasing N-glycans through the N-terminal site-selective succinylation assisted enzymatic deglycosylation. The obtained PGANs information is beneficial to not only achieve the deep coverage analysis of glycoproteomes, but also discover the new biological functions of such modification.

N-glycosylation is one of the most prevalent post-translational protein modifications^1^,^2^,^3^, which plays an important role in many biological processes, such as cell–cell interaction, protein folding and immune response^4^,^5^,^6^. In common N-glycoproteome studies, the advanced enrichment techniques, coupled with multidimensional chromatographic separation and high-resolution mass spectrometry (MS), have dramatically enhanced the dynamic range and limit of detection for N-glycosylation sites mapping^7^. However, the large-scale profiling of intact N-glycopeptides in complex samples remained a challenge with current technologies^8^. Therefore, in general MS-based approaches, the attached glycan needed to be removed prior to MS analysis, because the glycan part is favorably fragmented during CID, leaving the peptide part largely intact, thus hindering the identification^7^.

Several enzymes have been successfully developed for cleaving N-linked glycans, such as peptide-N-glycosidase F (PNGase F), endoglycosidase F and H^9^,^10^, among which PNGase F has emerged as a widely used glycoamidase due to its board substrate specificity and high activity. However, for peptides with glycosylated Asn at N-terminus (PGANs), the amide bond between the N-linked oligosaccharide chain and the glycosylated Asn residue is difficult to hydrolyze by PNGase F since the enzyme does not recognize peptides carrying N-terminal N-glycosylation^11^,^12^, making such sites extensively neglected in current glycoproteomic studies. Even though PNGase A has a broader substrate spectrum, the difficulty in recombinant expression and glycoprotein itself made it rarely used in current glycoproteomic studies^13^.

To address this problem, herein, we presented a strategy by incorporating succinylation at the N-terminus of PGANs for improving the efficiency of enzymatic deglycosylation catalyzed by PNGase F. Through the applications in the analysis of complex samples, the number and frequency of identified PGAN were obviously increased, promoting the comprehensive understanding of glycoproteomes.

## Results and Discussion

### Workflow for deep-coverage N-glycopeptide profiling

**Figure 1:**
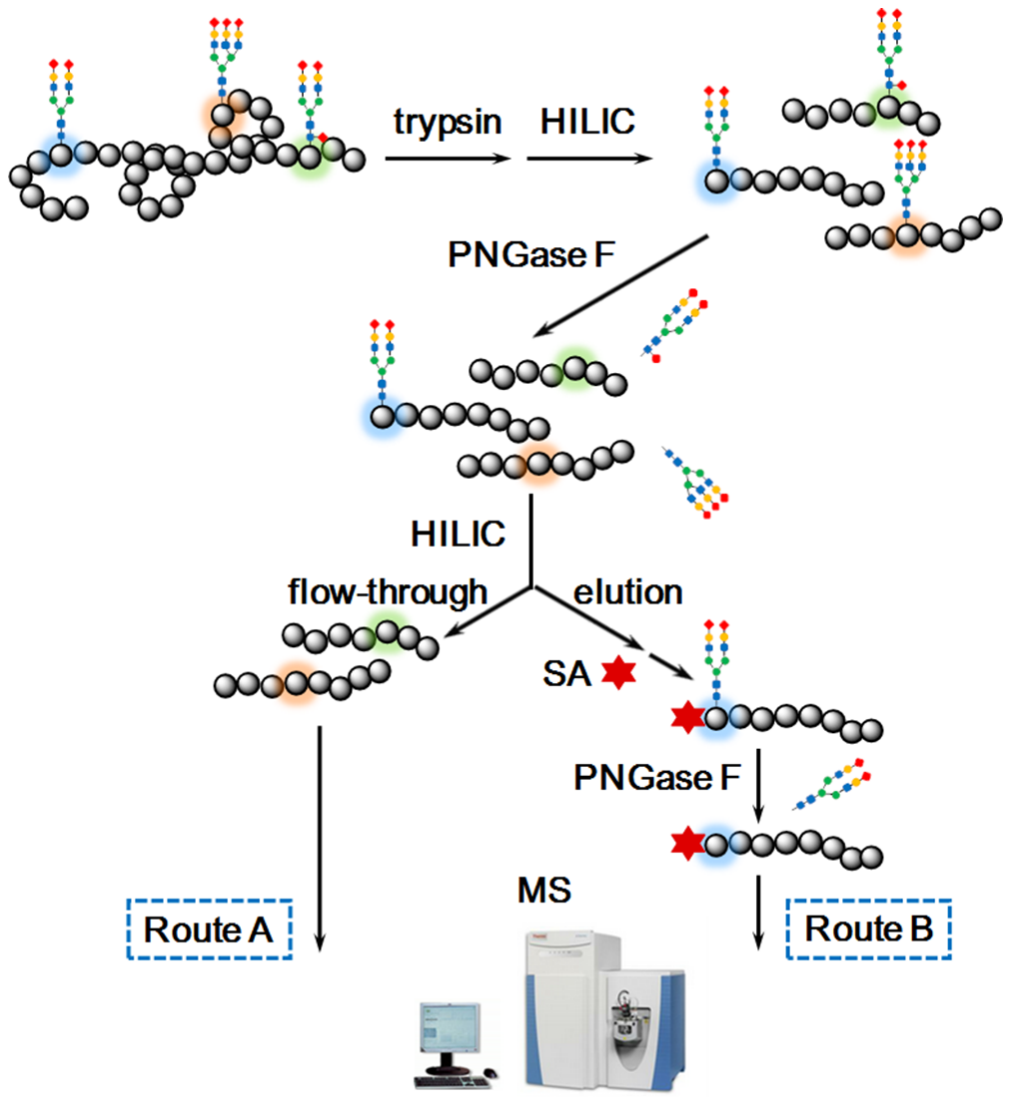
Flowchart of N-glycopeptides profiling with combination of commonly applied protocol (Route A) and our proposed protocol (Route B). The photograph of computer equipment was kindly provided by Y.J.W.

As shown in Fig. 1, firstly, the glycopeptides in protein tryptic digests were enriched by a hydrophilic interaction chromatography (HILIC) column packed with click maltose modified matrix^14^,^15^. After deglycosylation by PNGase F, most N-glycans were released, but N-glycans located at peptide N-terminus were still intact. In Route A, the deglycosylated peptides flowed through the HILIC column were collected for nano-LC-MS/MS analysis. In Route B, PGANs resistant to PNGase F were re-captured by HILIC, followed by labeling with succinic anhydride (SA) at the N-terminus. Finally, the labeled PGANs were further deglycosylated by PNGase F and analyzed by nano-LC-MS/MS.

### Evaluation on N-terminal succinylation assisted enzymatic deglycosylation

The N-glycopeptides from the tryptic digests of Ribonuclease B (RNase B), a glycoprotein with a single N-glycosylated site at Asn-60 exclusively occupied with known glycans varying from Man_5_GlcNAc_2_ to Man_9_GlcNAc_2_^16^, were used to evaluate our proposed strategy. Herein, SA was used to label PGANs, since it could be site-specifically attached to the peptide N-terminus by ring-opening reaction^17^. The peptide, QEPERNECFLSHKDDSPDLPK (one peptide originated from BSA digests), which contained abundant nucleophilic amino acids, such as Cys, Ser and Lys, was used to perform the optimization experiments in 50 mM phosphate buffer (PB, pH 8.0). As shown in Fig. S1, the poor labeling efficiency was obtained when the low concentration of SA (<10 mM) was used, insufficient for N-terminal succinylation. When the concentration of SA was increased to 40 mM, the labeling was also incomplete because of the acidic buffer. When 20 mM SA was used, all the peptides were N-terminal succinylated and only a few ε-amino groups at lysine (8.7%) were labeled. In addition, the reaction buffer was compatible with subsequent enzymatic deglycosylation, and the labeling process could be finished within 5 min.

**Figure 2:**
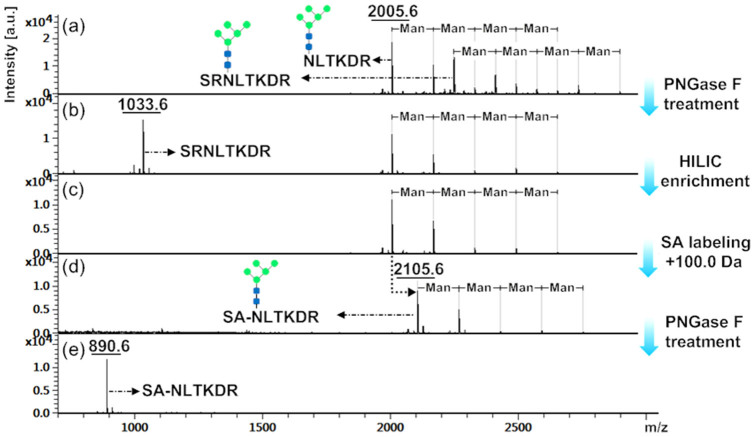
MALDI-TOF mass spectra of (a) N-glycopeptides enriched from RNase B tryptic digests, (b) peptides treated by PNGase F, (c) PGANs enriched by HILIC, (d) PGANs labeled with SA, and (e) deglycosylated and SA labeled PGANs.

The glycopeptides and their labeled products (10 μg) were detected by MALDI-TOF MS. As shown in Fig. 2a, two series of glycopeptides from RNase B digests were identified with peptide sequences of N_60#_LTKDR and SRN_60#_LTKDR, respectively (see Supplementary Fig. S2 and S3 online). Among them, each series contained five kinds of N-glycans with an equal mass difference (162.1 Da) derived from a mannose (Man). After treatment with PNGase F for 12 h, as shown in Fig. 2b, besides PGANs, glycopeptides with the peptide sequence of SRN_60#_LTKDR were totally deglycosylated, leaving a deglycosylated peptide with m/z = 1033.6 (see Supplementary Fig. S4 online), and PGANs with the peptide sequence of N_60#_LTKDR remained intact. Then the PGANs were re-enriched by HILIC as shown in Fig. 2c. After labeling with SA as shown in Fig. 2d, over 99% PGANs were succinylated, resulting in a mass shift of +100.0 Da. Finally, the labeled PGANs were incubated with PNGase F for 12 h and analyzed by MALDI-TOF MS. As shown in Fig. 2e, PGANs were successfully deglycosylated, leaving an SA labeled and deglycosylated peptide corresponding to SA-N_60#_LTKDR (m/z = 890.6) (see Supplementary Fig. S5 online). All these results proved that the selective N-terminal succinylation is favorable for releasing the N-glycan from PGANs by enzymatic deglycosylation.

### Mechanism of succinylation assisted enzymatic deglycosylation

PNGase F contains a Cys-His-Asp catalytic triad for hydrolyzing N-glycosidic bond^18^, and this active site is in close proximity to the glycosylated Asn during deglycosylation process^19^. The same as most enzymatic reaction, the N-glycopeptide substrate should be pre-stabilized before PNGase F catalyzes the deglycosylation. In this process, the Ser or Thr in canonical N-!P-S/T (where !P denotes any amino acid except proline) sequence can form hydrogen bonds with the peptide-binding channel of PNGase F^20^,^21^. It can be anticipated that the N-terminus of Asn should also be bound to PNGase F, which could enhance the stability of enzyme-substrate complex. If glycosylated Asn is located at the peptide N-terminus, the stability of enzyme-substrate complex is decreased, resulting in the unfavorable release of N-glycan.

To demonstrate our hypothesis, dimethyl ((CH_3_)_2_-N-) and butyraldehyde (-CH = N-) were introduced at the N-terminus of PGANs, respectively, but the labeled PGANs could not be recognized by PNGase F (see Supplementary Fig. S6 online). In contrast, with the above-mentioned succinylation (-CO-NH-) and the acetylation (CH_3_CO-NH-) (see Supplementary Fig. S7 online) introduced at the N-terminus of PGANs by succinic anhydride and acetic anhydride, respectively, the glycan at N-terminus of PGANs could be effectively released. All these results demonstrated that introducing an amido linkage at N-terminus of PGAN was crucial for increasing the stability of enzyme-substrate complex and hence greatly accelerated the enzymatic deglycosylation catalyzed by PNGase F.

### Performance of N-glycosylation sites mapping in HeLa cell

**Figure 3:**
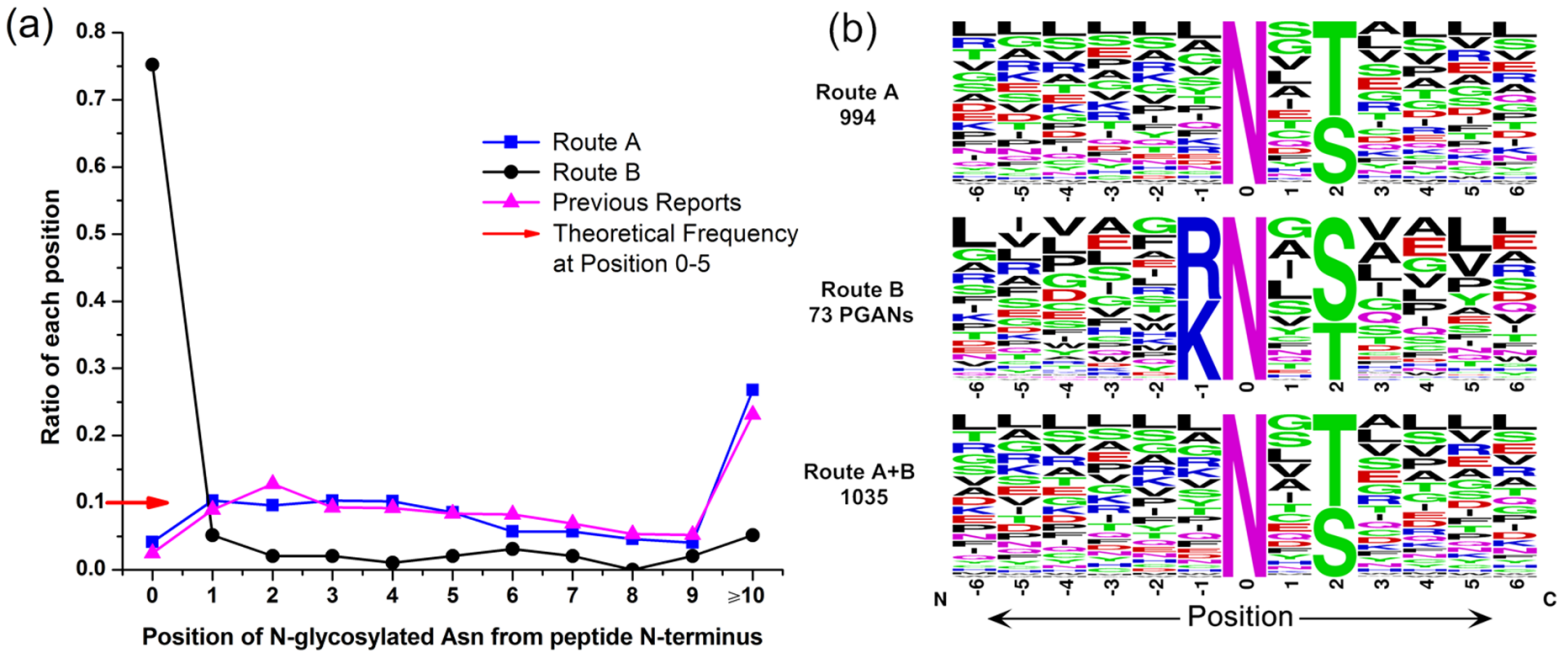
(a) Ratios of N-glycopeptides with modified Asn residues located at different position from peptide N-terminus, *i.e.* the position 0 stands for the glycosylated Asn located at N-terminus. (b) Distribution of non-redundant sequence motifs obtained by different routes.

This strategy was further applied to the N-glycoproteome analysis of HeLa cell lysate. To improve the confidence in N-glycosylation site assignment, the deglycosylation was performed in presence of H_2_O^18^ resulting in a mass shift of +2.9890 Da^1^,^22^. In our studies, the N-glycopeptides were identified for the peptides not only contained the sequence of N-!P-S/T, but also had the modification of deamination (+2.9890 Da) at the above Asn residue. In Route A, a total of 1135 unique N-glycopeptides were identified by three independent LC-MS/MS analyses (see Supplementary Table S1 online). Among them, PGANs only accounted for 4.1% of all glycopeptides (see Supplementary Table S2 online), much less than other glycopeptides (~ 10%) and theoretical frequency (~ 10%) (Fig. 3a). These results were also consistent with the previously reported results^23^. In Route B, in total, 97 unique N-glycopeptides were identified and as expected, more than 75% of them (73/97) were PGANs (see Supplementary Tables S3 and S4 online). All these PGANs were selectively labeled with SA at the N-terminal α-amine group (73/73, 100%). The low percentage of identified PGANs in Route A was mainly attributed to the poor activity of PNGase F for PGANs. In addition, we found that the large N-glycan positioned next to the Arg/Lys proteolysis site had minor hindrance for recognition by trypsin because the missed cleavage sequences of –KN- and –RN- (14.6%) were a little higher than other sequences, which also leads to the low percentage of PGANs. In addition, the carbamylation at peptide N-terminus and ε-amino group of lysine was investigated and no significant change of N-glycopeptides and PGANs was found. We further checked the succinylation occurred at other nucleophilic amino acids, and found that only 10% of the identified peptides were succinylated at Lys, Ser, Cys, Thr and Tyr. Therefore, the highly selective identification of PGANs in a proteome sample was achieved by site-specific N-terminal succinylation followed by enzymatic deglycosylation.

In Route A, 994 non-redundant sequence motifs were obtained by WebLogo^24^. As shown in Fig. 3b, Thr occurred more frequently (1.4-fold) than Ser at the second position, consistent with the previous reports^1^,^25^. The proportion of glycopeptides with Arg and Lys (blue font) at the -1 position was low and often ignored, mainly because that most PGANs were not deglycosylated, and hence failed for identification. As for the 73 PGANs identified in Route B, the -1 position was either Arg or Lys because the peptides were generated by tryptic digestion. With the combination of the sequence motifs in Route A and B, the proportion of N-glycopeptides with Arg and Lys at the -1 position was significantly increased (see Supplementary Tables S5 and S6 online). The overlap between PGANs identified in Routes A and B was also investigated (see Supplementary Fig. S8 online) and only 28.8% of PGANs in Route B can be overlapped, indicating these newly identified PGANs were necessary supplement for the glycopeptides identified by traditional approaches. However, 47 PGANs could be identified in Route A where SA was not used. To answer this question, we analyzed the spectral count (SC) and score of PGANs identified in both Route A and B. As expected, the average SC and score of PGANs identified in Route A (2.02, 53.02) were much less than those identified in Route B (3.63, 64.87), indicating that the majority of PGANs could be deglycosylated and identified with high confidence when SA derivation was used. Based on the overlap of identified PGANs via Route A and Route B, we classified PGANs for direct deglycosylation by PNGase F in three types, easy, medium and hard deglycosylation. As shown in Fig. S8, the sequence motif of N-!P-T tends to be easier for deglycosylation, consistent with previous research that a Thr at position +2 of Asn improved the activity of PNGase F compared with that of the Ser-containing glycopeptide^12^. In total, 99 PGANs were identified with high confidence, which accounts for 8.3% of all N-glycopeptides, much higher than those obtained by traditional approaches (59, 2.5%; 48, 3.6%)^23^,^26^.

Importantly, among the 73 PGANs confidently identified in Route B, 13 N-glycosylation sites were not annotated by UniProt (release 2014_09) and MaxQB (Version 4.2.2) database. For example, two membrane proteins (JNK1/MAPK8-associated membrane protein, K.N_#47_GSTEIYGECGVCPR, Tetratricopeptide repeat protein 17, K.N_#704_ISGALEAFR) and two secreted proteins (Heat shock 70 kDa protein 13, R.N_#184_STIEAANLAGLK, Collagen alpha-1(V) chain, K.N_#176_VTLILDCK) were identified as novel N-glycoproteins, which further demonstrated the feasibility and necessity of our proposed method.

### Performance of N-glycosylation sites mapping in mouse brain

**Figure 4:**
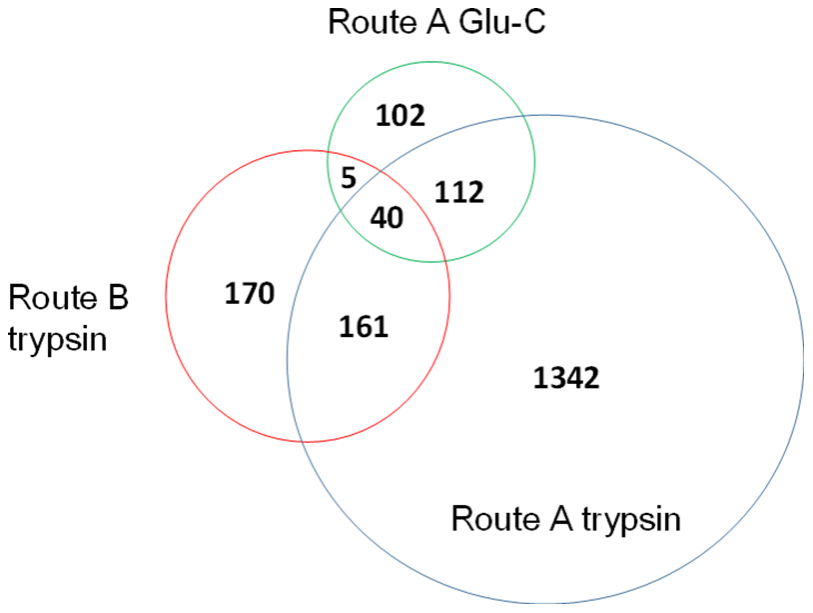
Overlap of identified N-glycosylation sites in mouse brain using three methods.

To demonstrate the advantages of our proposed method, trypsin and Glu-C were used to hydrolyze the equal proteins extracted from mouse brain in parallel, followed by analysis with LC-MS/MS (with Q-Exactive MS as the mass analyzer, see Supplementary Table S7). For tryptic digests, in Route A, 1869 N-glycopeptides (1655 N-glycosylation sites) were identified by three independent LC-MS/MS analyses, and PGANs only accounted for 4.5% (84/1869). In Route B, 397 N-glycopeptides (376 N-glycosylation sites) were identified by our succinylation assisted approach and 69.3% of the N-glycopeptides were PGANs, indicating most PGANs can only be identified when SA was used for labeling. Using Glu-C digestion method (named as Route A Glu-C), 311 N-glycopeptides (259 N-glycosylation sites) were identified by three independent LC-MS/MS analyses. The overlap of identified N-glycosylation sites by the above three methods was shown as follows in Fig. 4, the N-glycosylation sites obtained via both the succinylation assisted approach (Route B trypsin) and Glu-C approach provided supplementary information for trypsin results (Route A trypsin). By Route B trypsin, the number of N-glycosylation sites was increased by 10.3%, a little better than that obtained by the Glu-C approach (6.5%). It is worth noting that the overlap between succinylation assisted approach (Route B trypsin) and Glu-C approach (Route A Glu-C) was only 7.6% (45/590), indicating that such two approaches were complementary for extending the profiling of N-glycosylation sites.

Furthermore, the one-step deglycosylation approach (Route C) in which N-glycopeptides were succinylated before a single PNGase F cleavage was also performed (see Supplementary Fig. S9 online and Table S8). By contrast, by Route C, 1268 N-glycopeptides were identified. The percentage of PGANs was 12.8% (163/1268), much lower than the combined Route A and Route B. For Route C, with increased complexity of peptides, the succinylation for peptides was less complete and specific, compared to Route B, resulting in the decreased identified number of both N-glycopeptides and PGANs. Therefore, our proposed two-step strategy was much more advantageous than the one-step strategy.

In summary, to facilitate PNGase F based deglycosylation, herein, a site-specific amide linkage was proposed to incorporate the N-terminus of PGANs by labeling them with SA. Such labeling has been proved to be crucial for the identification of PGANs in proteome samples, which improved the comprehensiveness of N-glycosylation sites mapping. Furthermore, this work provides a novel way to fully exert the function of enzyme by modifying the structure of substrate.

## Methods

### Chemicals and materials

Ribonuclease B (RNase B, from bovine pancreas), trypsin (from bovine pancreas), Glu-C (from staphylococcus aureus V8), protease inhibitor cocktail, formic acid (FA), urea, trifluoroacetic acid (TFA), succinic anhydride (SA), acetic anhydride (AA), butyraldehyde, dithiothreitol (DTT) and iodoacetamide (IAA) were bought from Sigma-Aldrich (St. Louis, MO). Acetonitrile (ACN, HPLC grade) was purchased from Merck (Darmstadt, Germany). 2, 5-Dihydroxybenzoic acid (DHB) was obtained from Bruker (Daltonios, Germany). PNGase F was bought from New England Biolabs (Ipswich, MA). Water was purified by a Milli-Q system (Millipore, Milford, MA). All other chemicals and solvents were analytical-grade.

Fused-silica capillary (150 μm i.d. × 375 μm o.d.) was obtained from Sino Sumtech (Handan,China). Click maltose modified matrixes (5 μm, 100 Å) were kindly donated by Prof. Xinmiao Liang (Dalian Institute of Chemical Physics, Chinese Academy of Science, Dalian, China). Daiso C_18_ particles (5 μm, 100 Å) were ordered from Daiso (Osaka, Japan).

### Sample preparation

HeLa cells were cultured in DMEM medium containing 10% fetal bovine serum (FBS), and maintained in a humidified 37°C incubator with 5% CO_2_. Cells with nearly 90% confluence were harvested and washed with cold phosphate-buffered saline (PBS) for 3 times. Cell pellet was collected by centrifugation, and then suspended in 8 M urea (1% (v/v) protease inhibitor cocktail). Cell suspension was ultrasonicated on ice for 240 s in total (10 s intervals every 10 s). Samples were kept on ice for 2 min after sonication and centrifuged at 20,000 rpm at 4°C for 30 min. The supernatants were collected and the protein concentration was determined by the BCA assay. All percentages represented the volume percentage unless otherwise specified.

RNase B (dissolved in 8 M urea) and proteins extracted from HeLa cells were reduced in 20 mM DTT at 56°C for 1.5 h, and subsequently alkylated in 50 mM IAA at room temperature for 40 min in the dark. The solution was diluted with 25 mM NH_4_HCO_3_ (pH 8.0) to decrease the concentration of urea below 1.5 M. Then, trypsin was added at 1:30 (enzyme/substrate, m/m) and incubated at 37°C for 20 h. After 2 μL FA was added to end the digestion, the digests were desalted by a C_18_ precolumn and finally dried down in a Speed Vac Concentrator (Thermo, Waltham, MA). All samples were stored at −80°C pending further analysis.

Particularly, for proteins from mouse brain, the urea solution was diluted with 50 mM PB (pH 8.0) to decrease the urea concentration below 0.8 M. Then, Glu-C was added at an enzyme/substrate ratio (m/m) of 1:20 and incubated at 37°C for 20 h. Other conditions were the same as described above.

### Enrichment and deglycosylation of glycopeptides

The enrichment was performed similar to that described before^27^. Briefly, the HILIC column (4.6 mm i.d. × 1.0 cm) was packed with click maltose modified matrix. The tryptic digests (500 μg) extracted from HeLa cells or mouse brain were firstly dissolved in ACN/H_2_O/TFA (80:20:0.1) (buffer A) and loaded onto the HILIC column. Then, the column was rinsed with buffer A at 1.0 mL/min for 10 min. The glycopeptides were eluted with ACN/H_2_O/TFA (50:50:0.1), and re-dissolved in NH_4_HCO_3_ (50 mM in the presence of H_2_O^18^, pH 8.0). Subsequently, the glycans were released by adding 500 units of PNGase F and incubated at 37°C for 12 h. Finally, the peptides were desalted and dried for further use.

### Re-enrichment, labeling and further deglycosylation

The deglycosylated products were re-enriched by HILIC column as described above. The deglycosylated peptides and glycopeptides were collected in loading buffer and eluting buffer, respectively. The former was analyzed by nano-LC-MS/MS (Route A). The latter (PGANs) were re-dissolved in phosphate buffer (PB, 50 mM in the presence of H_2_O^18^, pH 8.0) containing 20 mM SA. After 5 min reaction at room temperature, the labeled peptides were deglycosylated by adding 100 units of PNGase F and incubated at 37°C for 12 h. Finally, the deglycosylated peptides were desalted for nano-LC-MS/MS analysis (Route B).

For the glycopeptide from RNase B digests, the deglycosylation was performed in the presence of H_2_O^16^ and followed by MALDI-TOF MS analysis. Other conditions were the same as described above.

### MS analysis

MALDI-TOF MS analysis was performed on Ultraflex III TOF/TOF (Bruker Daltonics, Germany) with constant laser intensity in positive ion mode. DHB matrix solution (20 mg/mL) was prepared in ACN/H_2_O/H_3_PO_4_ (50:50:0.1). One microliter of the sample and DHB solution were orderly spotted on the plate for MS analysis.

The deglycosylated peptides from HeLa cell lysate were analyzed by 1D nano-RPLC-MS/MS on a Triple TOF 5600 plus (AB SCIEX, USA) MS. The mobile phases were 2% ACN with 0.1% FA (phase A) and 98% ACN with 0.1% FA (phase B). A C_18_ trap column (150 μm i.d. × 5 cm) was connected to a homemade capillary separation column (75 μm i.d. × 15 cm). For separation, a 130-min gradient was established, comprised of 100 min of 8%–20% B, and then 30 min of 20%–28% B, with the flow rate at 280 nL/min. The mass spectrometer was operated in positive ion data dependent mode with one MS scan followed by 60 MS/MS scans using a 22 seconds exclusion window. The scan range of full MS was set from m/z 350 to m/z 1500, while the MS/MS was set from m/z 100 to m/z 1500.

The deglycosylated peptides from mouse brain were analyzed by 1D nano-RPLC-MS/MS on a Q-Exactive MS (Thermo Fisher Scientific, USA) equipped with an Ultimate 3000 (Dionex, USA) nano LC system. The mobile phases and separation column were the same as described above. A 100-min gradient was established for separation. The spray voltage was 2.7 kV and the temperature of ion transfer capillary was set at 275°C. The Q-Exactive was operated in positive ion data dependent mode with one MS scan followed by 15 MS/MS scans using a 20 seconds exclusion window. MS1 was performed at the resolution of 70,000, ranging from m/z 300 to m/z 1800 (automatic gain control (AGC) value: 1e6, maximum injection time: 100 ms). MS2 was performed at the resolution of 17,500 (AGC: 1e5, maximum injection time: 50 ms).

### Database searching

For the datasets produced on Triple TOF 5600 plus MS, the acquired *.wiff and *.wiff.scan files were converted to *.MGF files by PeakView (version 1.2.0.3) and searched against the human IPI database (version 3.87) in Mascot (version 2.3.2). Mass tolerances for Triple TOF 5600 plus were set as 0.1 Da for parent ions and 0.05 Da for fragments. For the datasets produced on Q-Exactive MS, the *.raw files were converted to *.mgf by Proteome Discoverer (version 1.4.0.288) and searched against the UniProtKB mouse complete proteome sequence database (release 2014_11). Mass tolerances for Q-Exactive were set as 7 ppm for parent ions and 20 mmu for fragments. Reverse sequences were appended for FDR evaluation. Cysteine carbamidomethylation (+57.0215 Da) was searched as a fixed modification. Oxidation (M) (+15.9949 Da), acetylation (protein N-termini) (+42.0106 Da) and deamidation (N) (H_2_O^18^) (+2.9890 Da) were searched as variable modifications for Route A. For Route B, the succinylation (+100.01 Da) of Lys and peptide N-terminus were appended as variable modifications. To investigate the carbamylation derived from urea, carbamylation (N-termini and Lys) (+43.0247 Da) were also added as variable modifications for both routes. Peptides were searched using tryptic cleavage constraints, and up to 2 missed cleavages. For Glu-C digestion, the missed cleavage was set as 4. The search results were filtered by pBuild (version 2.0) to control the FDR ≤ 1%. The N-glycosylation sites were identified if the peptides not only contained the canonical sequence of N-!P-S/T (where !P denotes any amino acid except proline), but also had the modification of deamination (+2.9890) at Asn.

## Author Contributions

Y.J.W., H.J., L.F.C., L.H.Z. and Y.K.Z. designed research; Y.J.W. performed most of the experiments; Z.S. and Z.G.S. helped to perform the experiments; Y.J.W. and Y.C.S. analyzed data. Y.J.W., Z.G.S. and L.H.Z. wrote the manuscript. All authors reviewed the manuscript.

## Supplementary Material

Supplementary Information Supplementary Information

Supplementary Information Dataset 1

Supplementary Information Dataset 2

Supplementary Information Dataset 3

Supplementary Information Dataset 4

Supplementary Information Dataset 5

Supplementary Information Dataset 6

Supplementary Information Dataset 7

Supplementary Information Dataset 8
