# Supplementary material for: Releasing N-glycan from Peptide N-terminus by N-terminal Succinylation Assisted Enzymatic Deglycosylation
Source: Sci Rep. 2015 Apr 22;5:9770. doi: 10.1038/srep09770 (PMC4405948; doi:10.1038/srep09770)
Supplement: Supplementary Information [file srep09770-s1.doc]

Supporting Information

Releasing N*-*glycan from Peptide N-terminus by N-terminal Succinylation Assisted Enzymatic Deglycosylation

Yejing Weng^1,2^, Zhigang Sui^1^, Hao Jiang^1,2^, Yichu Shan^1^, Lingfan Chen^1,2^, Shen Zhang^1,2^, Lihua Zhang*^1^ and Yukui Zhang^1^

^1^Key Lab of Separation Sciences for Analytical Chemistry, National Chromatographic R. & A. Center, Dalian Institute of Chemical Physics, Chinese Academy of Sciences, Dalian 116023, China. ^2^Graduate University of China Academic of Sciences, Beijing 100049, China.

*Corresponding Author.

Address: 457 Zhongshan Road, Dalian 116023, China

E-mail: [lihuazhang@dicp.ac.cn](mailto:lihuazhang@dicp.ac.cn)

Tel/Fax: +86-411-84379720

**Results**


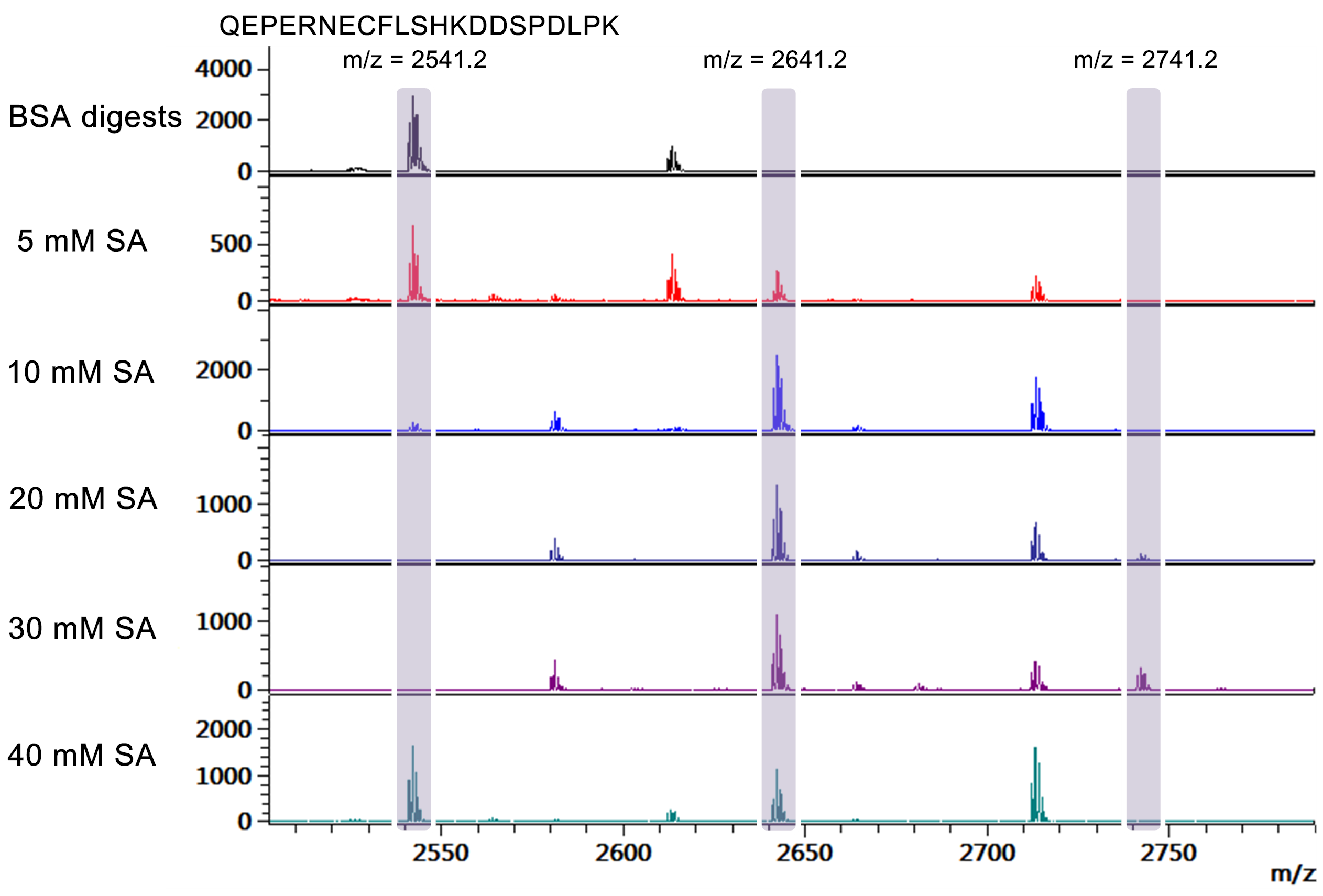


**Fig. S1** Optimization experiments for peptide N-terminal succinylation.


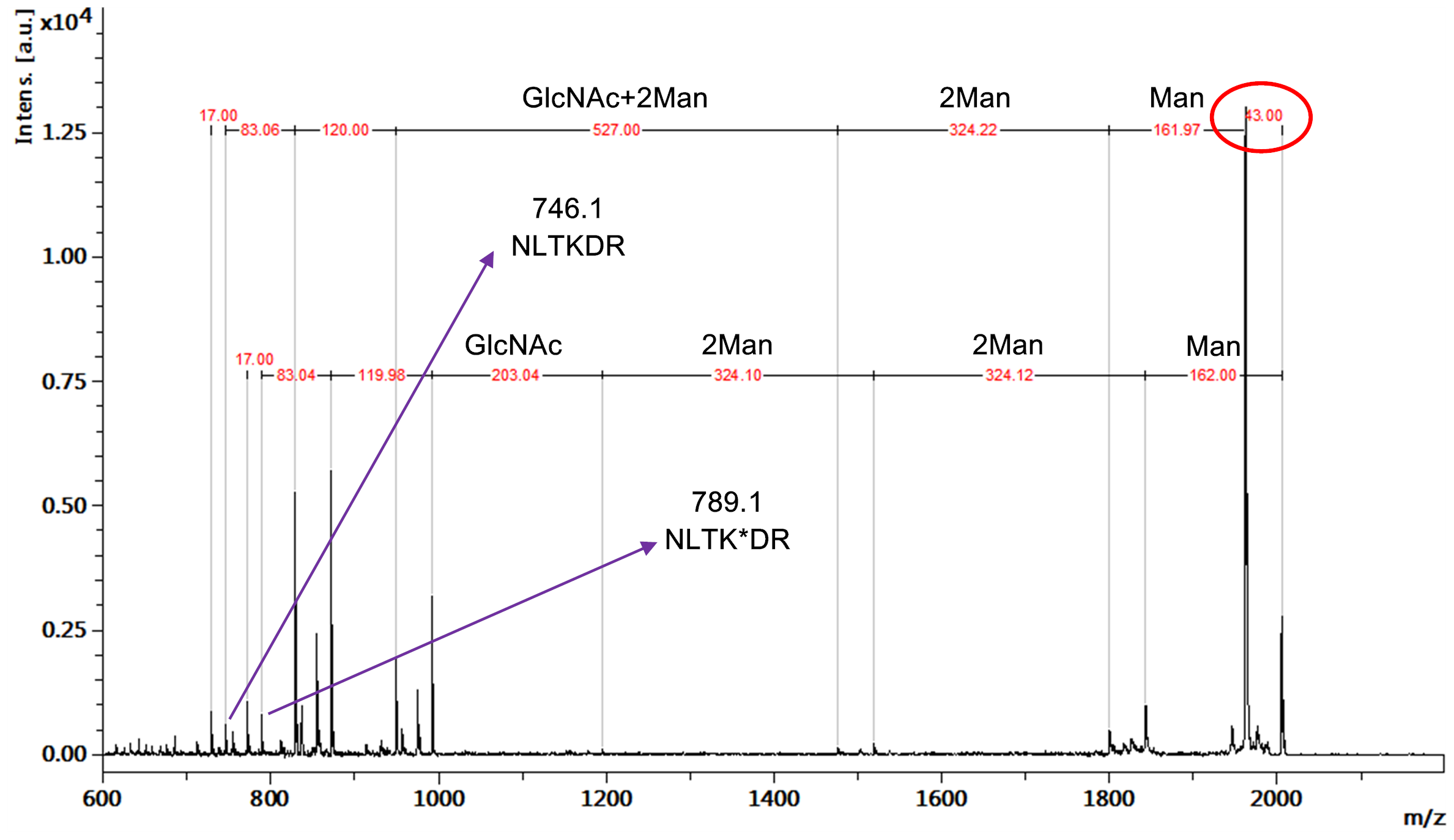


**Fig. S2** MS/MS spectrum of m/z 2005.6, attributing by N-glycopeptides of N_60#_LTKDR with the glycan of Man_5_GlcNAc_2_. The lysine (K) was carbamylated, and hence miss-cleaved by trypsin. The asterisk (*) stands for carbamylation modification, similarly herein after.


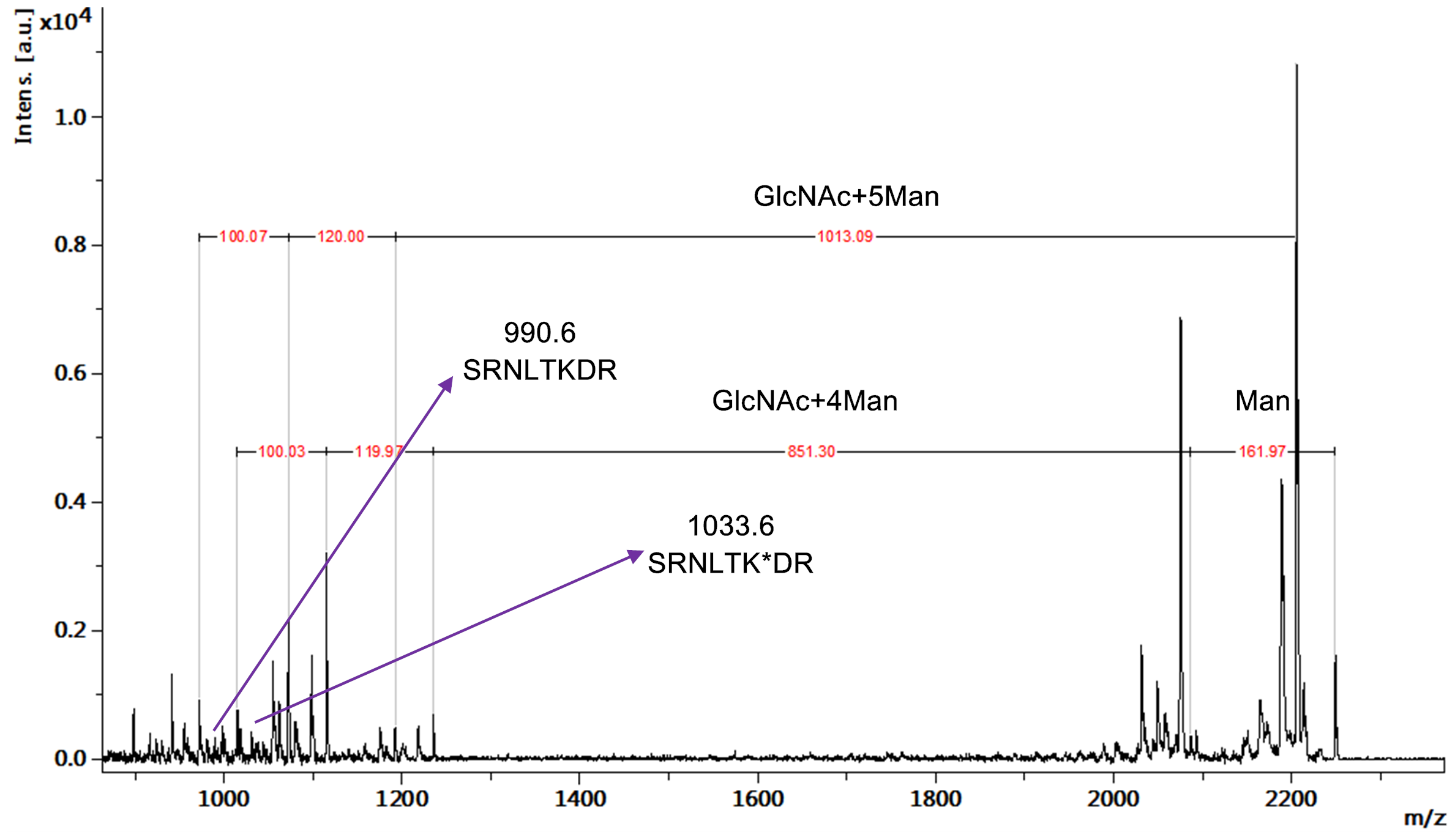


**Fig. S3** MS/MS spectrum of m/z 2248.6, attributing by N-glycopeptides of SRN_60#_LTKDR with the glycan of Man_5_GlcNAc_2_.


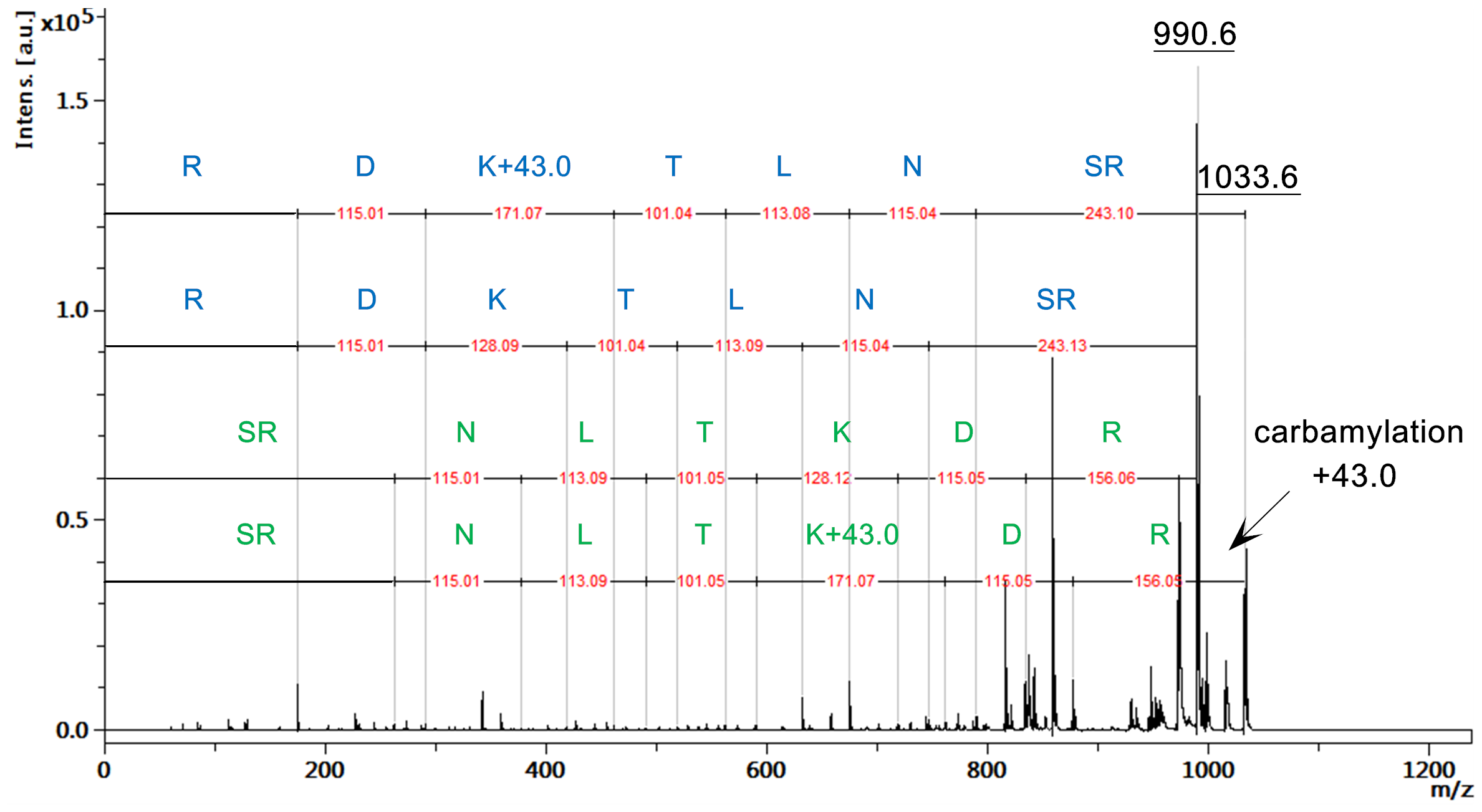


**Fig. S4** MS/MS spectrum of m/z 1033.6, attributing by the deglycosylated peptide of SRN_60#_LTK*DR.


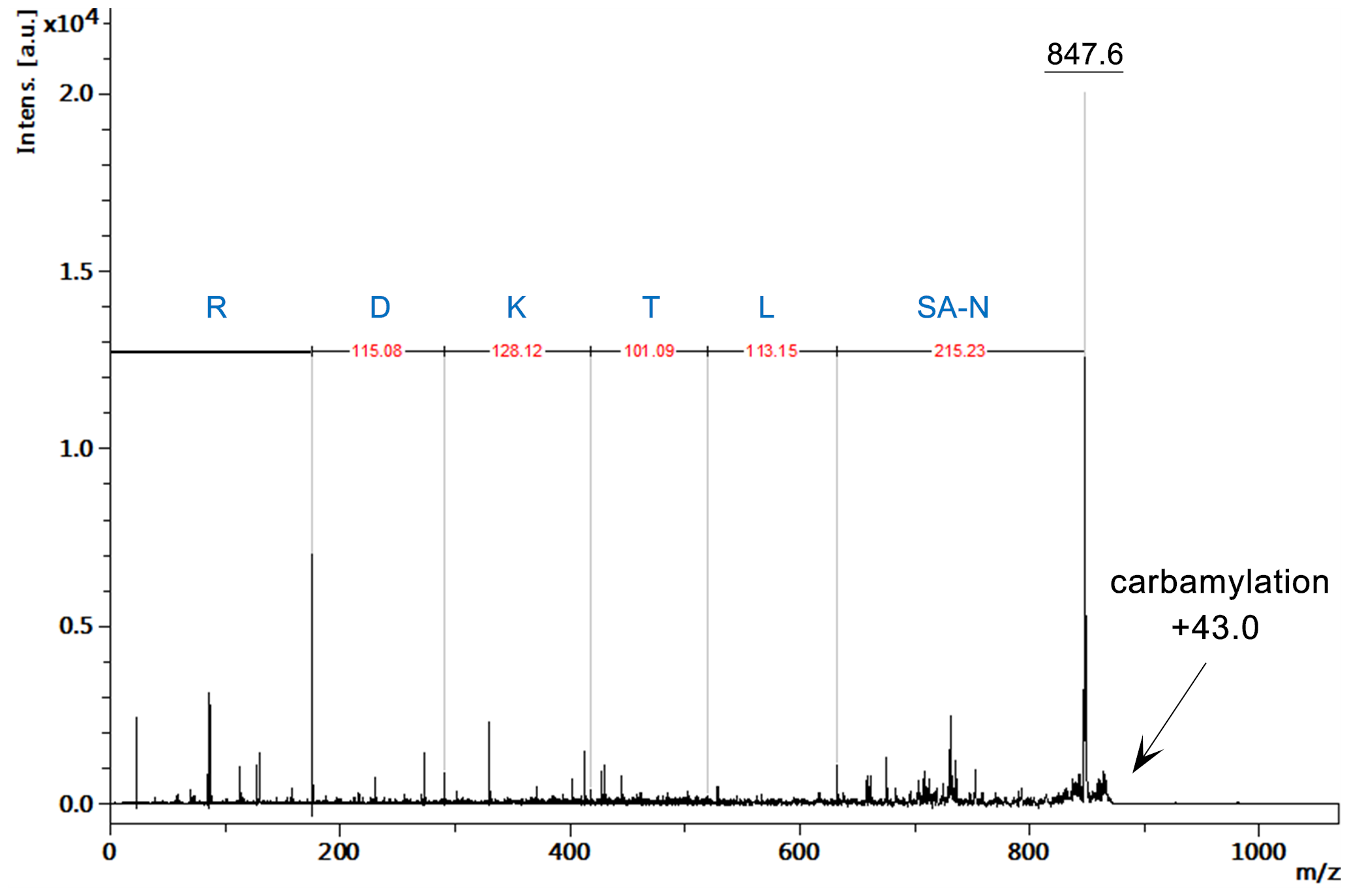


**Fig. S5** MS/MS spectrum of m/z 890.6, attributing by the SA labeled and deglycosylated peptide of SA-N_60#_LTK*DR.


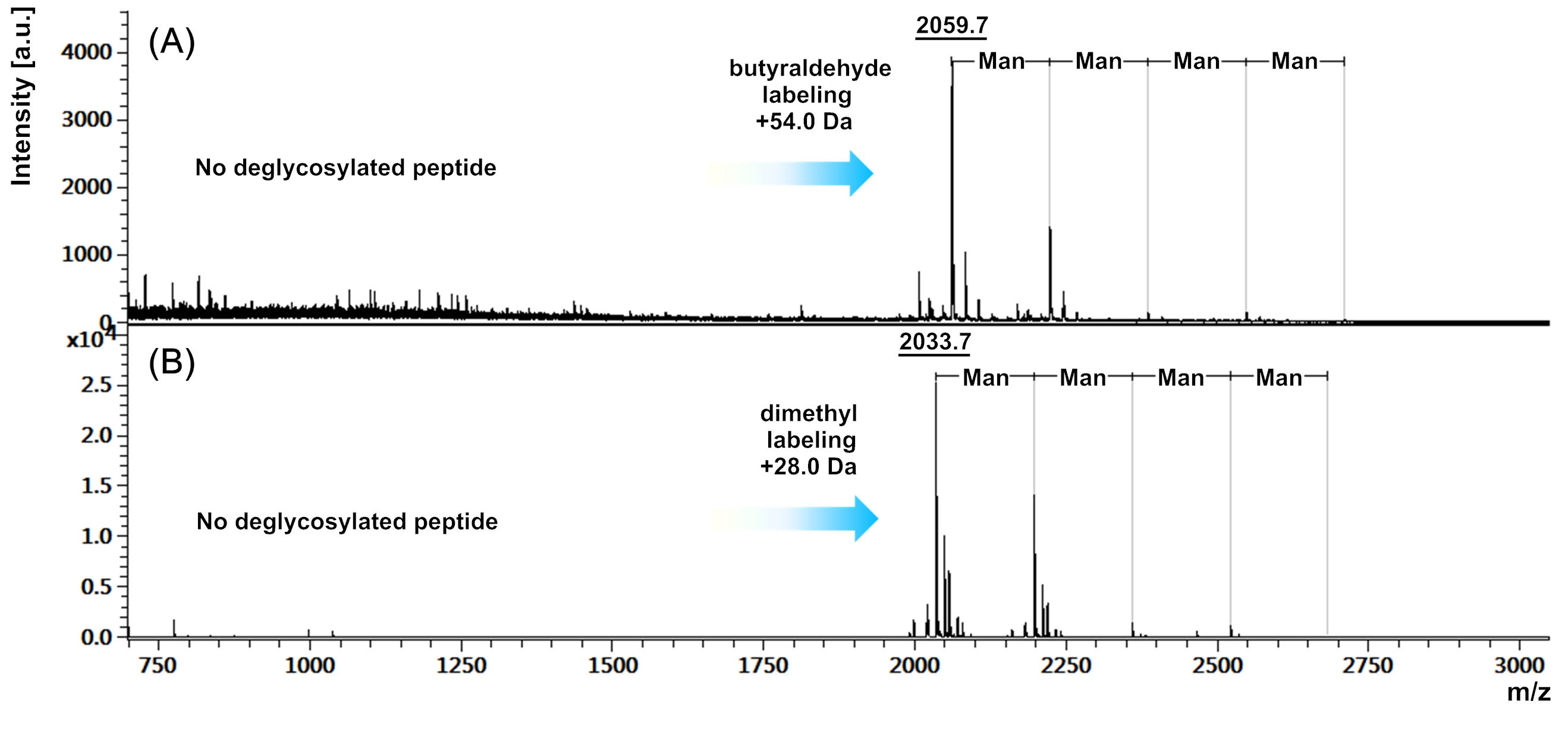


**Fig. S6** MALDI-TOF mass spectra of (A) butyraldehyde labeled PGANs after treatment by PNGase F (+54.0 Da) and (B) dimethyl labeled PGANs after treatment by PNGase F (+28.0 Da).


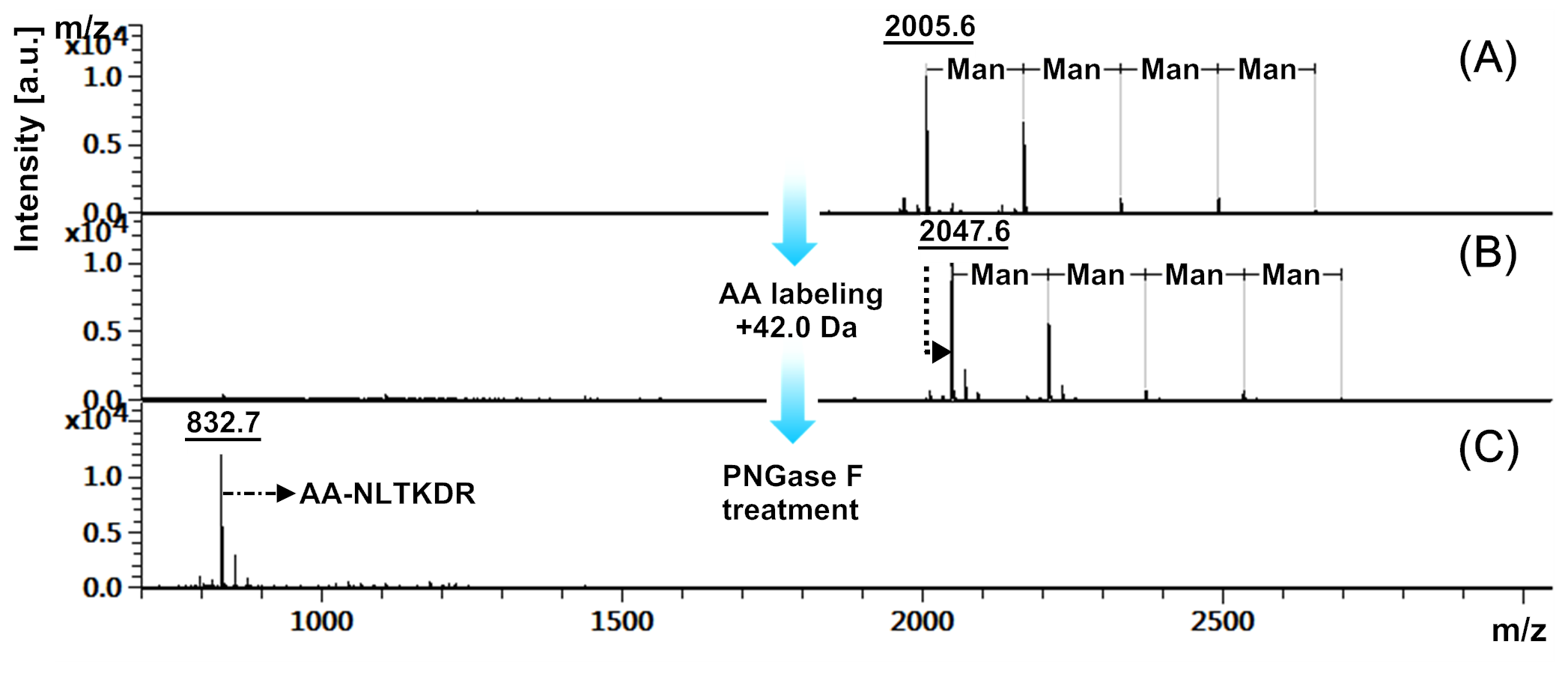


**Fig. S7** MALDI-TOF mass spectra of PGANs (A) enriched from RNase B tryptic digests (10 μg), (B) labeled with AA by N-terminal acetylation, and (C) AA labeled and deglycosylated by PNGase F.


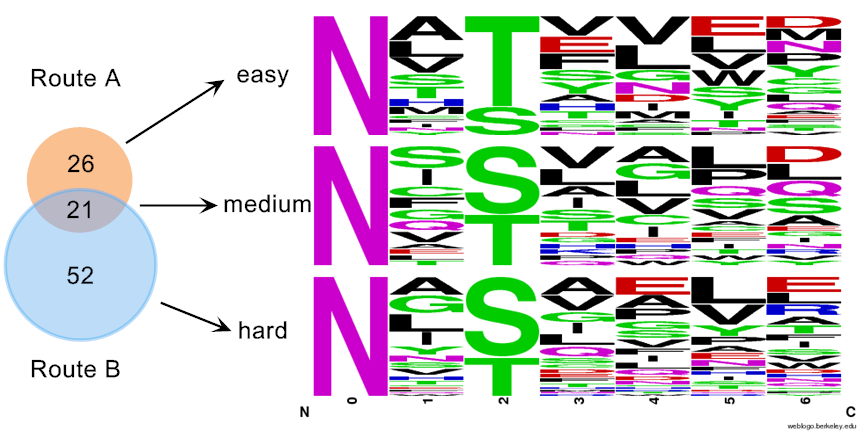


**Fig. S8** Classification of PGANs with three kinds of sequence motifs for direct deglycosylation.


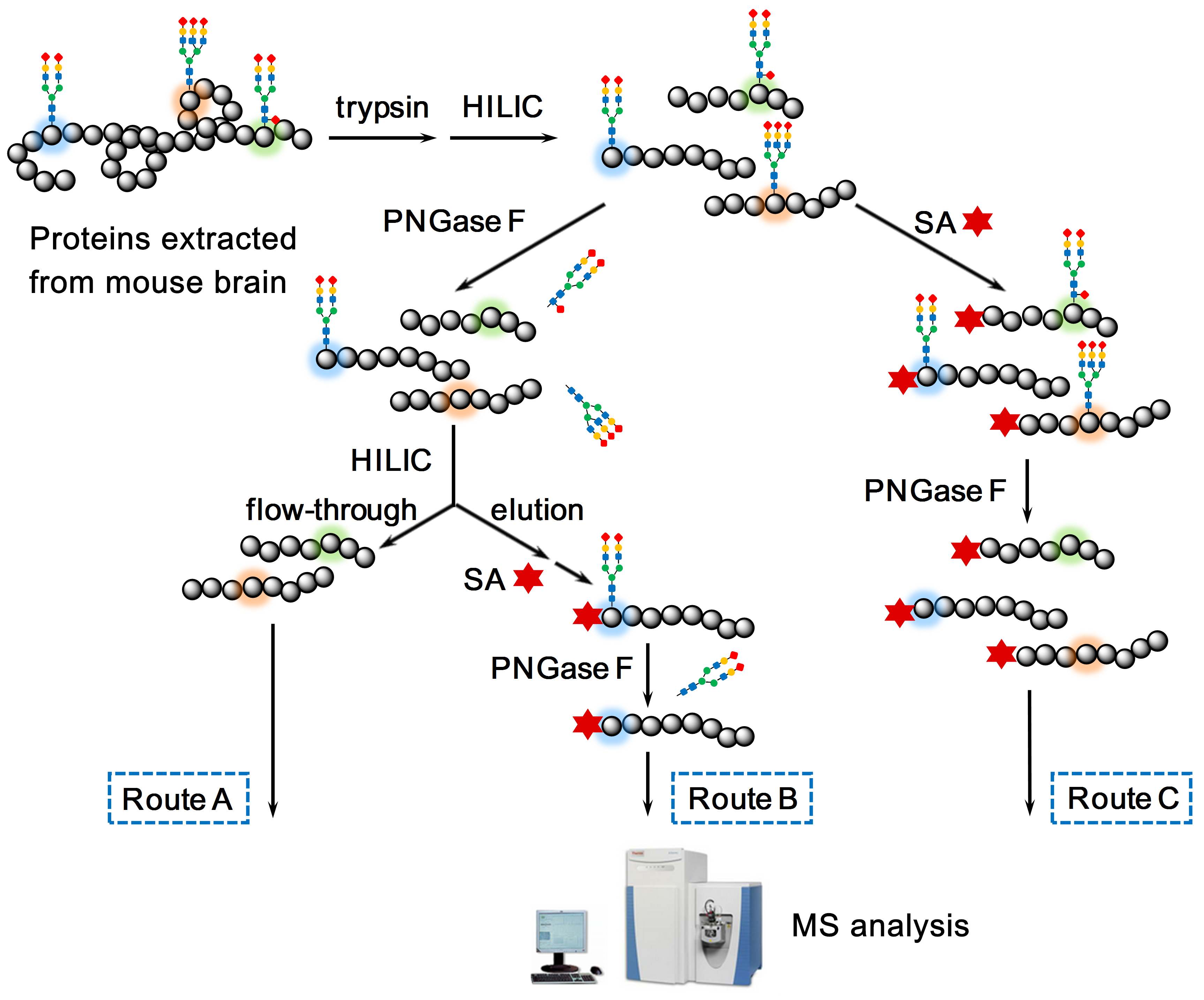


**Fig. S9** Flowchart of N-glycopeptides profiling by two-step routes (Route A and Route B) and one-step route (Route C). The photograph of computer equipment was kindly provided by Y.J.W.
